# Respiratory Diagnoses Year‐Round: Unraveling the Multifaceted Pediatric Infection Cycles

**DOI:** 10.1111/irv.70037

**Published:** 2024-11-04

**Authors:** Marcin Piotr Walkowiak, Jarosław Walkowiak, Dariusz Walkowiak

**Affiliations:** ^1^ Department of Preventive Medicine Poznan University of Medical Sciences Poznań Poland; ^2^ Department of Pediatric Gastroenterology and Metabolic Diseases Poznan University of Medical Sciences Poznań Poland; ^3^ Department of Organization and Management in Health Care Poznan University of Medical Sciences Poznań Poland

**Keywords:** pediatric, primary care, respiratory infections, seasonality, syndromic surveillance, tonsillitis

## Abstract

**Objective:**

The aim of the study is to analyze the annual cycle of pediatric medically attended respiratory illnesses.

**Study Design:**

Data on 141 million pediatric respiratory visits from the years 2010–2019 were obtained from the Polish National Healthcare Fund. To identify underlying patterns and trends within the aggregated data, techniques like seasonal‐trend decomposition using LOESS (STL) and principal component analysis (PCA) were applied.

**Results:**

A strongly recurring pattern was observed. Following the annual minimum in late summer, there was a sudden surge in upper respiratory infections in early September. Subsequently, overall visits declined gradually, while the share of lower respiratory infections increased, particularly during the influenza peaks from January to March. Afterwards, visits declined steadily, with an additional peak of tonsillopharyngitis noted in midsummer. Dimensionality reduction of diagnoses implied the existence of two major groups of co‐occurring diagnoses, the proportions of which change over the year: one smaller but more severe, peaking during the influenza season, and the second dominating with lower severity. Age differences in diagnoses were observed, with babies showing upper respiratory infections likely diagnosed with the common cold rather than a more specific upper respiratory infection.

**Conclusion:**

While enhancing surveillance strategies is indeed a desirable long‐term goal, it is worth noting that despite the variability observed in the onset of the influenza season, the infection cycles generally follow a relatively fixed pattern. This consistency provides a foundation for effective planning and underscores the potential for proactive measures to mitigate the impact of seasonal outbreaks.

## 1 Introduction

Despite the predictable pattern of increased medically attended respiratory illnesses among children every infection season, there is a surprising dearth of large‐scale population wide studies that could be utilized for the purpose of healthcare system planning and public health. It appears that current research on this subject is fragmented, with individual studies shedding light on specific aspects of the phenomenon.

Due to the increasing availability of PCR tests, numerous studies have been conducted on the prevalence and seasonality of specific viruses among patients. However, these studies often rely on data from hospitals [1, 2], emergency departments [3], or hotlines [4] skewing their sample towards the most severe and urgent cases. Consequently, they offer less information on milder cases that may only necessitate a visit to a family doctor. Moreover, studies including milder cases present an additional challenge—a higher share of patients turns out to be infected by unrecognized pathogens [2, 5], presumably those that were sidelined in research due to their low virality. This issue is further compounded by the fact that research on healthy individuals demonstrates a significant rate of asymptomatic infection [6].

There is a high number of studies on syndromic surveillance, although their intended role is to detect unique outbreaks of particularly virulent pathogens as they unfold. While they may provide insights into the typical infection season when numerous pathogens are simultaneously circulating, this aspect is typically addressed as a secondary consideration at best. Nevertheless, in order to pinpoint the prevalence of influenza‐like illness (ILI) symptoms, they have to filter out milder respiratory infections. Thus, they are able to show a cycle with the smallest number of medically attended infections in late summer, followed by a sudden increase in autumn, with a later increase in severe infection that could serve as a good predictor of incoming influenza waves [7].

Research on the seasonality of pediatric visits is limited and often addresses the issue indirectly. Lipsett et al. have documented an annual pattern in visits to the emergency department, which not only shows distinctive cycles for upper respiratory infections and pneumonia but also related cycles for otitis media and gastroenteritis [3]. The cycle of changing severity of pediatric respiratory infection was demonstrated by Astudillo et al. in their sample from Santiago de Chile [8]. Additionally, there are works addressing the subject indirectly by examining how weather and school openings influence the onset of the infection season [9] or by investigating the role of students in infecting their teachers [10]. Some studies have hinted at the already‐mentioned cyclical pattern while researching whether the etiology is viral or bacterial [11].

The aim of this study is to investigate the annual pediatric visit pattern in relation to infectious diseases, primarily for the purpose of healthcare system planning and public health. The objective is to deeply analyze relationships between age, diagnosis, cycle, variability, or their interrelationships within a large‐scale dataset. The goal is to detect less straightforward patterns that are clinically relevant or useful for healthcare resource utilization.

## 2 Methodology

### 2.1 Data Source

Data on the number of weekly diagnoses of acute respiratory infections (ARI) coded under ICD‐10 (J00–J22) for ISO‐weeks spanning from 2010‐W1 to 2019‐W52 were obtained from the Polish National Healthcare Fund which provides free universal coverage. The data, while inclusive of all provider submissions to the government system, overwhelmingly represent diagnoses made during primary care visits due to the nature of these conditions. To safeguard patient confidentiality, age was based only on the year of birth, and instances where the weekly diagnoses for a specific age group in an administrative district fell between 1 and 4 were represented as “< 5.” For computational purposes, this was assumed to be 2. Based on such criteria, there were 141 million pediatric visits for patients below 18 years of age in the analyzed period. Summary with annual number of particular pediatric visits was presented in supplementary materials.

### 2.2 Calculation

Codes used infrequently (less than 0.01% of visits) were grouped with their dominant counterparts; for example, a few cases of influenza with an identified virus were aggregated with the primarily used code for influenza with an unidentified virus. Moreover, the names were simplified for presentation purposes. Weekly sums were presented. Subsequently, the data were log‐transformed, cleaned of the trend component to remove impact of demographic fluctuations using the seasonal‐trend decomposition using LOESS (STL) model, the log transformation was reversed, and the data were equalized into a percentage deviation from the annual trend. For models that require a fixed‐length cycle, the ISO leap week 2015‐W53 was omitted. For presentation purposes, major consistently recurring interruptions are marked on the graph, such as the summer holidays (late June to the end of August), the Christmas Break (from Christmas Eve to New Year's, followed immediately by Epiphany), and the May bridge holidays (May 1 and 3). The exact duration of these interruptions varies depending on the day of the week the holiday falls and the type of educational institution, as schools generally have extended leave around official holidays, while preschool facilities tend scale down operations on days adjacent to holidays due to lower attendance.

To explore the overall structure of infection seasons, we applied principal component analysis (PCA) to the weekly diagnosis counts to identify patterns of co‐occurring diagnoses. The aim was to determine whether overlapping infection waves exhibit sufficiently different virulence to create distinctive patterns in overall diagnostic proportions. This analysis yielded two main epidemiological dimensions, whose components were further examined. Because the number of circulating pathogens cannot be negative, we adjusted the values of the calculated axes by adding their respective minimums, transforming them into nonnegative explanatory variables. These adjusted axes were then used as predictors in a linear regression model without an intercept.

In order to analyze the general structure of infection seasons and detect conditions that exhibit distinctive patterns, weekly numbers of diagnoses were subjected to PCA. Two major epidemiological dimensions were formed, and their composition was analyzed. Under the assumption that the amount of circulating pathogens could not be negative, these axes were transformed into explanatory variables by adding their respective minima to them and used as explanatory variables in a linear regression model without an intercept. Annual diagnosis cycles obtained from STL were plotted.

Statistical analysis and data visualization were conducted in Python 3.10 using default data processing libraries such as pandas 1.4.3 and NumPy 1.26.0rc1. Statsmodels 0.14.0 was used to calculate STL, while PCA and linear regression were performed using sklearn 0.0.post1. Data visualization utilized the plotly library version 5.14.1.

## 3 Results

**FIGURE 1:**
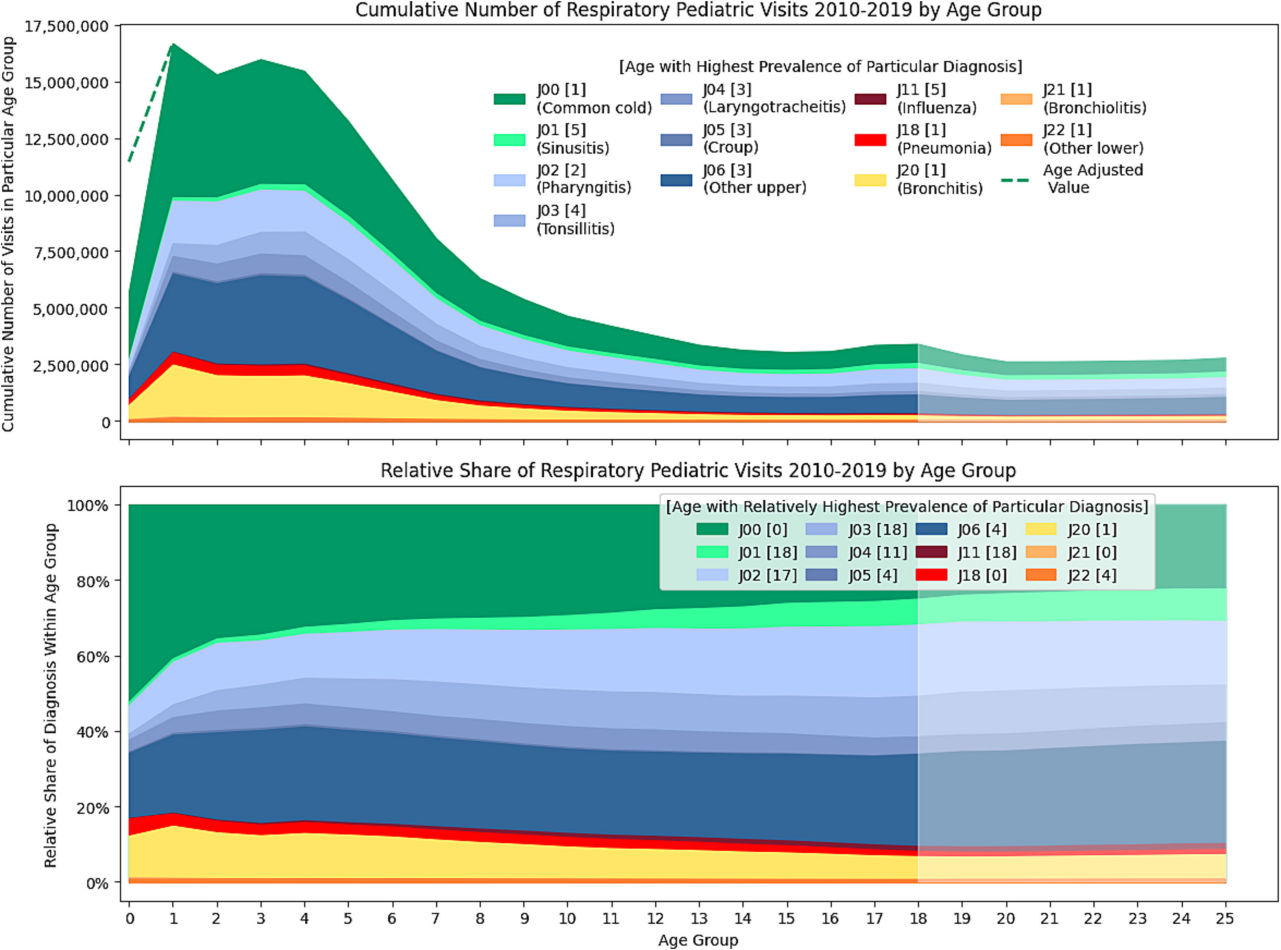
Cumulative number of respiratory pediatric visits by age group.

The pediatric visit data, categorized by age group, are depicted in Figure 1. It is important to note that age group 0 comprises infants born in a specific calendar year and should not be directly compared due to its increasing size throughout the year. Even with this adjustment, the visit count remains somewhat lower than the plateau observed for children in the 1–4 age group. Subsequently, there is an initial steep decline that gradually slows during early teens but subtly persists into early adulthood. Notably, a minor increase is evident for the 17–18 age group, which does not appear to be random noise, as a similar pattern was independently observed in Poland in COVID‐19 detection solely among girls of the same age [12] and may rather reflect nuanced healthcare utilization patterns. In nominal terms, the majority of diagnoses are most likely to peak among the youngest children. Interestingly, the diagnosis with the latest peak at fifth year is J11 (influenza), and this late peak appears more like a diagnostic issue, as seroprevalence studies suggest somewhat earlier contact [13, 14].

As presented in Figure 1, the younger the patient, the higher the probability that the diagnosis would be J00—acute nasopharyngitis (common cold), which is especially noticeable for infants. Nevertheless, based on the insignificantly reduced share of the most severe diagnosis in the age group 0, it does not appear to result from parents being more likely to seek medical help for their sick infants. The explanation could be twofold: In 1‐year‐olds, where the cycle could be analyzed, diagnoses of the common cold were consistently elevated but more evenly distributed, suggesting a potential first encounter with some mild viral strains that, in other age groups, might rarely cause symptomatic infections. Conversely, the observed relationship could simply be an artifact of the challenges in examining younger patients [15]. This is especially noticeable as with age increases, the share of J00 (nasopharyngitis) decreases, but the share of J01 (pharyngitis) increases. Similarly, the relative share of some more specific diagnoses like influenza actually increases with age, though the general share of more severe lower respiratory diagnoses subtly declines into early adulthood.

**FIGURE 2:**
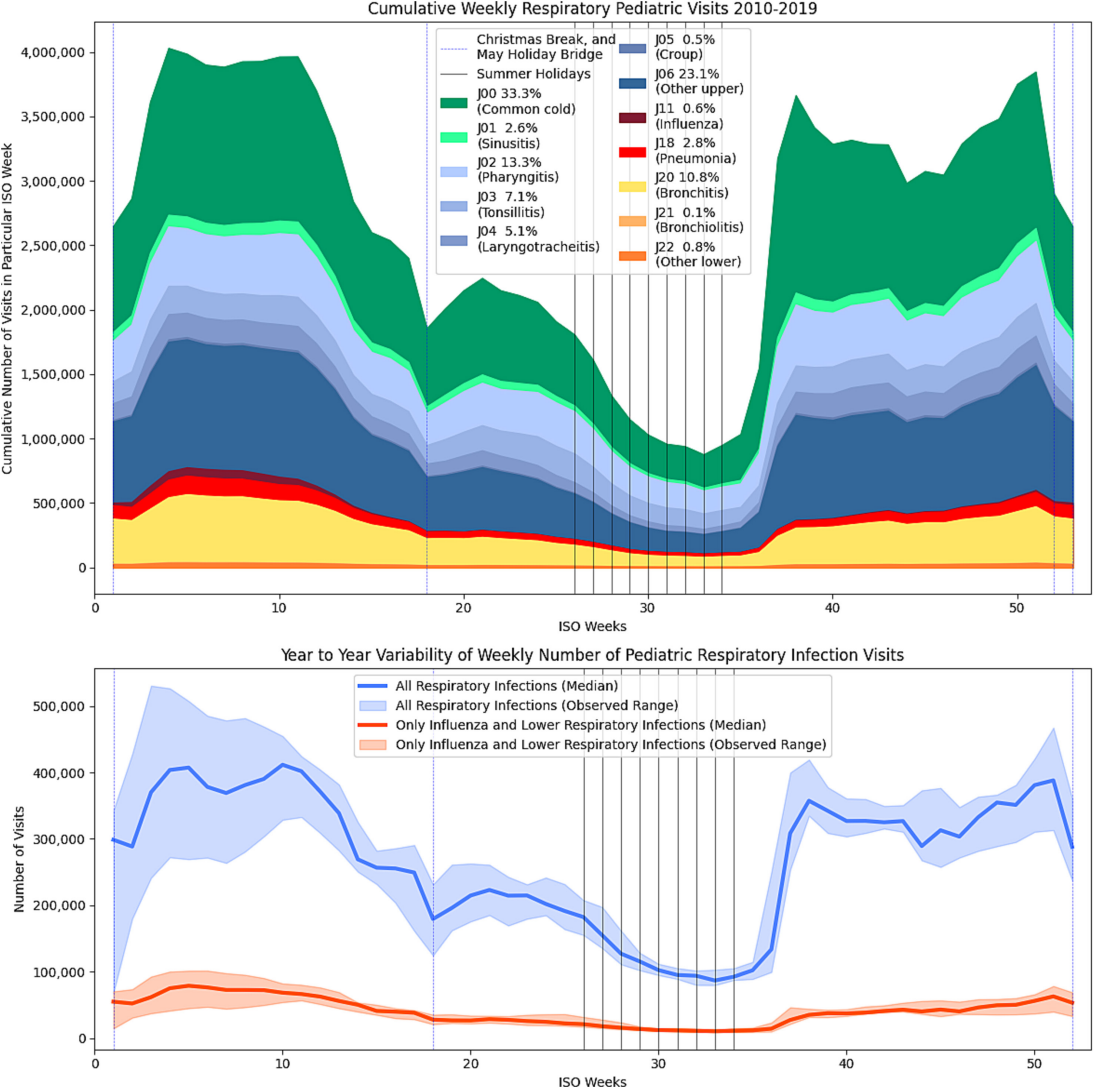
Total number of weekly visits for respiratory diagnoses.

The annual cycle of respiratory visits is plotted in Figure 2. The lowest number of infections is in Week 33, and it starts climbing even before the opening of schools in Week 35. In the next 2 weeks, the number of infections climbs to a peak in Week 37. From that moment, there is a less obvious pattern observed—the number of lower infections is slowly climbing, while the number of upper infections is steeply declining, creating a modest net decrease in the overall number of visits. Subsequently, the period from Week 50 to Week 15 is considered the influenza season. The plotted data show a relatively low number of influenza diagnoses during that period, accompanied by a high number of lower respiratory visits. This observation is attributed to the analyzed period occurring before the widespread issuance of suitable tests capable of differentiating ILI in the aftermath of the COVID‐19 pandemic [16]. Subsequently, through spring and summer, there is a steady reduction in the number of infections. The most stable period was late summer; conversely, the most unstable period was late winter, with declining variability within the second half of the influenza season.

On the graph, there are dips that do not reflect the epidemiological situation but result from limited availability during holidays. During those weeks, there is a significant reduction in milder upper respiratory infections, while only a minor reduction occurs in lower infections. This is especially noticeable at the end of the year (Christmas, New Year, and Epiphany) but also in Week 17, where there are two consecutive holidays in Poland.

**FIGURE 3:**
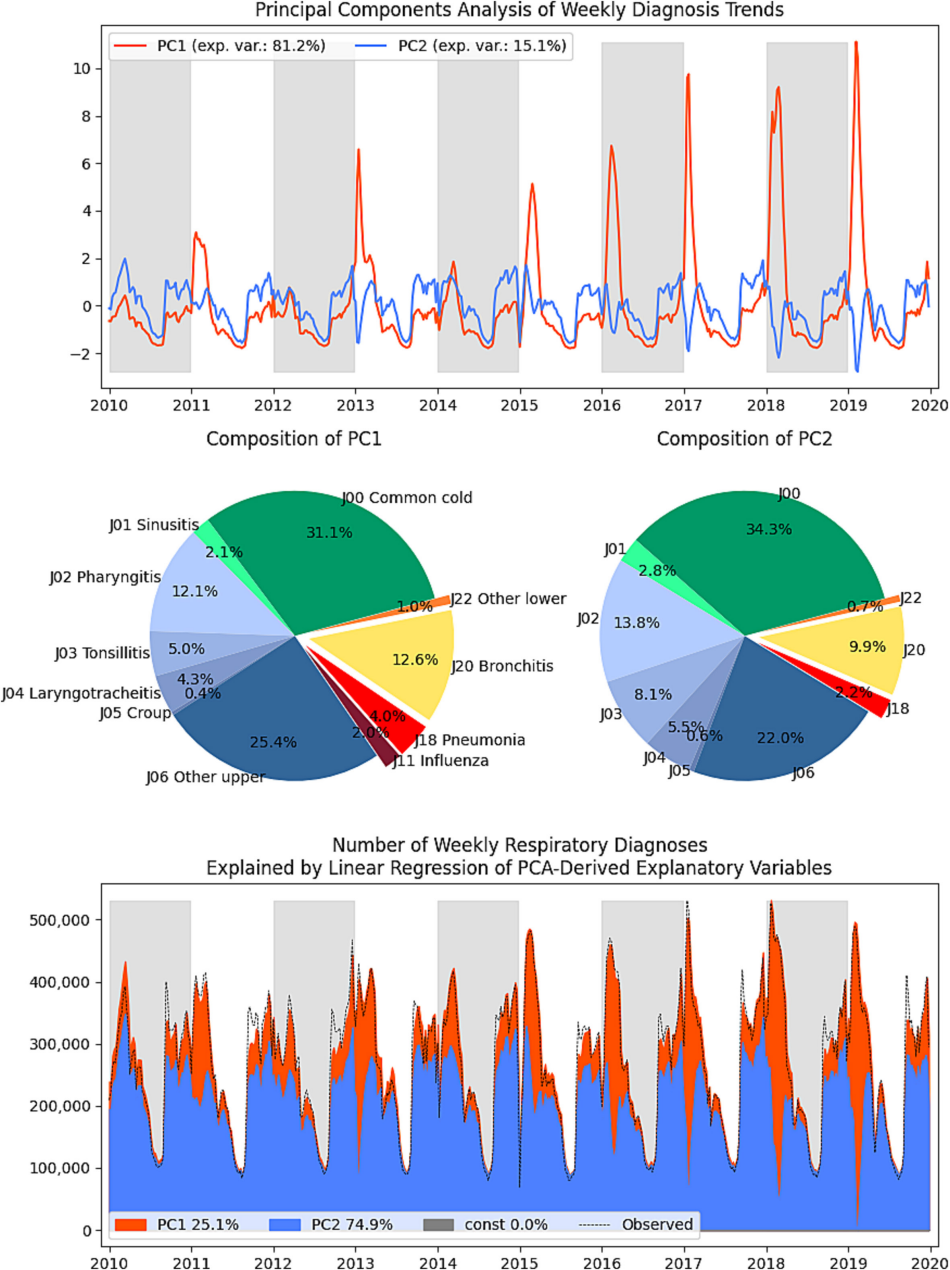
Principal component analysis of weekly diagnosis trends.

The results of PCA are presented in Figure 3, with two major axes explaining 96% of the variance. Based on the explained variance, the first axis appears to be dominant; however, it rather seems that it better represents variation in conditions that are more likely to lead to more severe outcome [17, 18]. When the axes were turned into explanatory variables for the linear regression model without an intercept, it was actually PC2 that explained approximately three times more doctor's visits. When the composition of these diagnoses is observed side by side, it may appear superficially similar. However, the share of pneumonia is 3.6% versus 2.0%, indicating a highly relevant difference, and a similar distinction is noticeable for any other lower respiratory infection. All PCA weights were positive, except for the negative weight of influenza in the second axis. These two axes were also consistently underestimating the number of peak infections in early autumn.

The PCA axes appeared to capture tangible patterns instead of being just mathematical constructs, as for both axes, PCA all weights were positive implying being product of more and less virulent pathogens, except for the negative weight of influenza in the second axis. This was noticeable on the graph, as during influenza peaks, the axis reflecting milder diagnoses appeared to have been crowded out.

**FIGURE 4:**
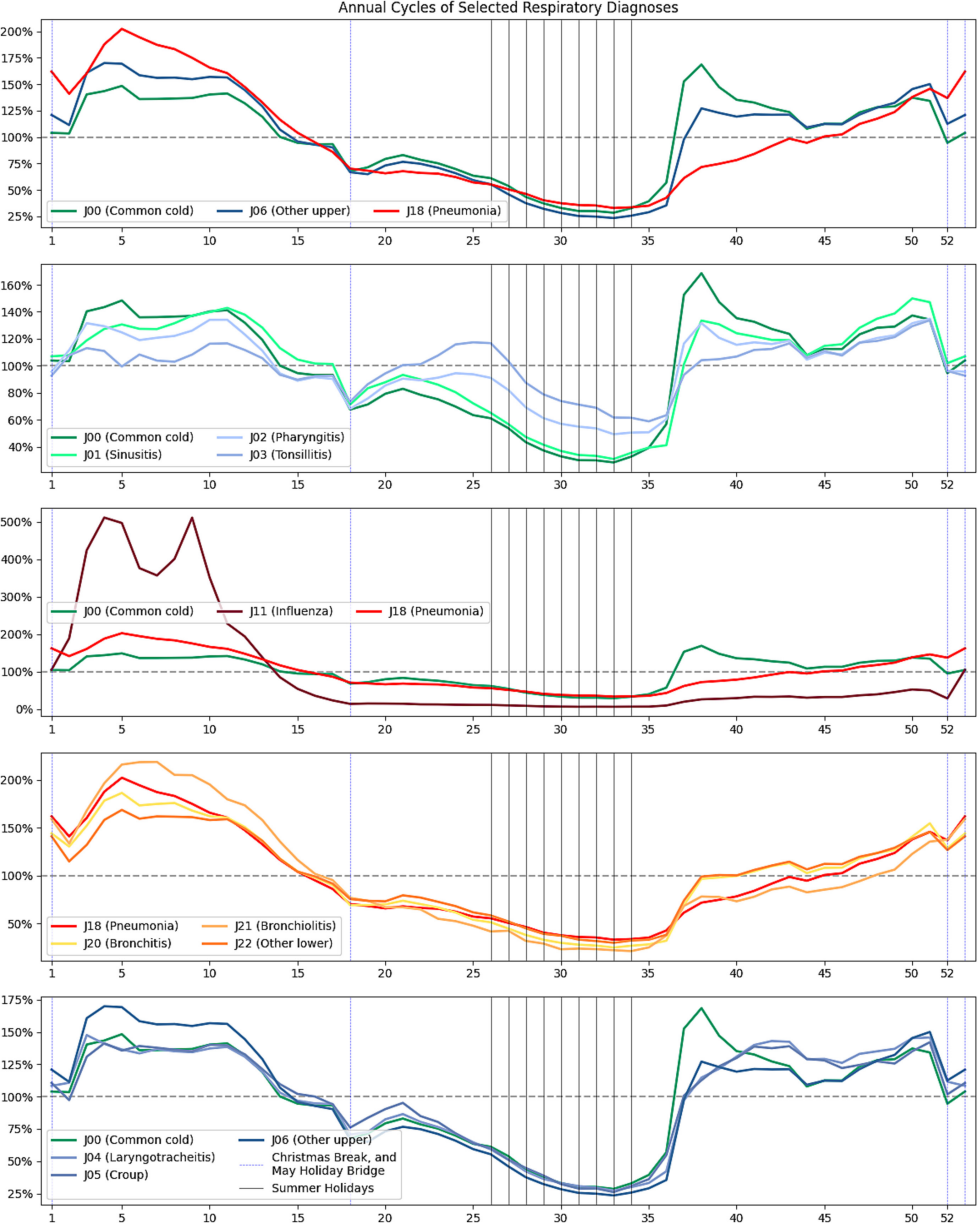
Annual cycles of selected respiratory diagnoses.

The overall cycle of infection was presented for selected diagnoses in Figure 4. All conditions are scaled with 100% being their average annual prevalence. There is a clearly different cycle with an almost immediate onset in early autumn for the common cold, while pneumonia has its peak amid the influenza season. Code J06 (acute upper respiratory infections of multiple and unspecified sites) had a cycle that manifested as an approximate interposition of those two cycles and appeared to be used both as shorthand for a nastier common cold and for ILIs. Influenza was the diagnosis with the highest annual variability, though its peak roughly matched the peak of lower respiratory infections, which among themselves had rather similar cycles. For pediatric patients, the model detected two independent influenza peaks instead of one for adults; however, this appears to be a result of the infection wave being regularly interrupted by winter holidays.

While upper respiratory infections otherwise had a substantial cycle overlap, there was one noticeable divergence, J03 (acute tonsillitis), and partially J02 (acute pharyngitis). Not only did these conditions have a clearly much flatter cycle than other respiratory infections, but they also had an additional infection wave independent from other cycles that occurred in the middle of summer, with a peak around Week 25. This unique infection wave was only noticeable for pediatric patients, as even among teenagers, this particular diagnosis was undergoing diminishing cyclical amplitude, suggesting that this particular respiratory infection is atypical and is only significantly influenced by circulating pathogens in pediatric patients.

## 4 Discussion

While there is a mechanism for how infectious diseases spread due to changes in weather conditions, contact patterns, and acquired immunity, with a significant amount of randomness such as immunity‐evading mutations or superspreader events, the stochastic mechanism forms a consistent annual pediatric visit pattern due to the high number of interposed outbreaks. After the annual minimum in late summer, there is a sudden surge in mild infections in early September. There is a slow decline in overall visits, though the severity gradually increases with influenza peaks occurring from January to March. Afterward, there is a slow, steady decline in visits, except for an additional peak of tonsillopharyngitis in midsummer. Based on diagnosis, the overwhelming majority of circulating strains could be classified into dominating less virulent ones and more severe ones that, during the influenza season, effectively crowd out diagnoses related to milder pathogens.

While the cycle of more virulent conditions clearly peaks during apparent peaks of influenza A, the mechanism appears to be more nuanced. Firstly, the plot clearly diverges from what one would theoretically expect from the infection wave following the susceptible–infectious–recovered (SIR) model [19], as the wave appears much more elongated than expected and often with local maxima. Thus, a more likely origin would be the superposition of more severe infection waves. From better monitored pathogens that appear to constitute this group, influenza A is likely preceded by an RSV wave which is especially affecting pediatric patients and superseded by influenza B [20], both known to cause lower but more elongated infection waves [21, 22]. Additionally, based on the viruses detected in tertiary hospitals [1] or those for which patients are more likely to seek medical help [23], this more virulent group is likely to include adenovirus and parainfluenza virus as well.

The apparent squeezing out of other infectious agents presented by the model most likely has a few sources of origin. Firstly, the data show that in case of limited access to medical help, there is a significant drop in the number of patients with milder diagnoses while only modest with more severe ones, and a similar process is highly likely during system overload at the peak of influenza waves. Secondly, PCR studies to determine the etiology of infectious agents are likely to detect coinfections, especially among pediatric patients. Thirdly, there is a well‐established competitive relationship among respiratory viruses, with the influenza season reducing the positivity rate of human rhinovirus (HRV) even among asymptomatic patients [6] and both HRV and endemic coronaviruses among surveyed households [23]. However, sole influenza epidemics with coinfection and competition are highly unlikely to explain this axis on their own, as even during peaks of influenza epidemics, the share of people influenza positive among those who show symptoms of ILI barely exceed 50% during influenza peak [24]. Thus, it is also likely that the model found a mathematical fit that explains the observed data well, though instead of creating an axis of the most virulent viruses in circulation, it created an axis capturing the most virulent observed composition. This would explain the still relatively high share of relatively mild diagnoses within this axis.

To understand the composition of the analyzed axis, it is also necessary to examine milder infections that might not otherwise draw attention. Almost half of otherwise healthy infants in the Lee et al. study were technically asymptomatic, with the predominant diagnosis being HRV, though the likelihood of detecting viruses from that group was highest among infants with mild symptoms [6]. In the Bonfim et al. study on children attending daycare, who underwent onsite medical assessments for mild respiratory concerns—primarily presenting with a running nose but no fever—the dominant diagnosis often indicated an absence of detectable viruses, with HRV being the most frequently identified when a virus was present [5].

Monto et al. also illustrate an interesting pattern of underrecognition. In their study, participants were instructed to collect swab tests even for milder symptomatic infections. In instances where HRV, unknown pathogens, or endemic coronaviruses jointly constituted the dominating diagnoses, fewer than 1/5 of cases prompted patients to seek medical help. However, this probability doubled in cases involving more virulent strains like influenza [23]. On one hand, this suggests that the second axis in the model encompasses not inherently less virulent infections but rather a subset of less virulent infections that nevertheless prompted individuals to seek medical help, introducing a selection bias that overestimates its severity but underestimates its prevalence. Additionally, in studies analyzing these mild patients, there was a notable share of more severe strains detected. This somewhat aligns with the findings of Pierangeli et al., who, on a relatively small sample of emergency department patients, demonstrated that not only the majority of cases with ILI were not caused by influenza but also the majority of laboratory‐confirmed influenza cases did not even exhibit ILI symptoms [25].

The initial autumn peak appears to be initially primarily driven by HRV, and consistently with the change in the number of visits, studies on this pathogen's prevalence suggest that the number of infections begins to rise even before the start of school year September [6] though this virus remains present throughout the season and could even cause an additional spring wave [23]. Additionally, it includes endemic coronaviruses that have their distinctive season peaking in January and February [26] and unidentified pathogens, the nature of which can only be analyzed indirectly as they reveal themselves through the infections they induce. The fact that a significant share of circulating viruses eludes detection is consistent with the relatively mild nature of their infections, which attracts limited scientific inquiry. However, as research on subtypes of HRV or endemic coronaviruses was detailed enough to detect differences in virality between their major subgroups [6, 27], it suggests that the undetected viruses likely share characteristics making their detection more challenging, such as high heterogeneity or an even higher prevalence of asymptomatic cases, introducing ambiguity in discerning their pathogenic nature.

Apart from diagnoses aligned with the annual cycles of low temperature–modulated viral infections, tonsillitis and, to a lesser extent, pharyngitis deviated with lower annual amplitudes. Additionally, both conditions displayed an unusual rise in midsummer of unclear etiology. While there are some rare cases of viruses, such as enteroviruses, having a summer season [28, 29], the more likely suspect would be bacteria, which tend to thrive in warmer conditions. Nevertheless, Group A Streptococcus, typically associated with these diagnoses, can be excluded as a major factor in this infection wave. Studies show either erratic, location‐specific infection patterns [30] or that a subtle summer increase in pharyngotonsillitis is accompanied by a drop in this pathogen's positivity rate [31]. Suspicion on seasonal changes in the composition of the etiology of those conditions is further supported by somewhat different prognosis, as cases of peritonsillar abscess are generally more likely in warmer seasons [32, 33, 34].

Most findings appear to be closely related to epidemiological phenomena observed in other countries, as for example the major cycle of pediatric visit show eerie similarity to described already in 1986 by Ayres annual fluctuations of bronchitis diagnosis in the United Kingdom [35]. Annual patterns of both milder and more severe pathogens closely resemble those presented by Schrijver et al. among pediatric outpatients and inpatients of a teaching hospital in the Netherlands [2]. The fluctuations in respiratory tract infections aligned with our data on severe infection, while the changes in asthma cases ‐ appearing as lingering outcomes of mild viral circulation [36, 37], the authors surprisingly attributed to “school stress.” However, they lacked an elegant explanation for why they detected a minor epidemic of “school stress” in early autumn, a more relaxed period in late autumn, and then a secondary, even more stressful period in the aftermath of the influenza season. Additionally, this “school stress” was manifesting primarily as asthma, while visits due to behavioral problems failed to produce statistically significant fluctuations [2].

The findings are largely generalizable due to the convergence in epidemic timing in Europe [38]; however, a few caveats and adjustments should be considered. Despite significant year‐to‐year variability, there is a subtle underlying pattern showing that in the northern hemisphere, the higher the latitude, the later the peak of the RSV and influenza season [39]. Tracing symptomatic differences in diagnoses is likely to be biased through nuanced differences in coding conditions, and even some subtle regional inconsistencies were noticeable within this single country dataset, especially in cases where the distinction would not be clinically significant. There are notable variations in medical help‐seeking behavior across Europe. For instance, around two‐thirds of Belgians with ILI sought a doctor's assistance immediately, while among Swedes, only about one‐fourth sought help, mostly when the symptoms persisted for a week [40]. While the findings of this study would need to be recalibrated to local patterns of medical help‐seeking behavior and diagnostic coding practices, identifying that symptomatic diagnoses show two distinctive axes would allow for further refinement of symptomatic surveillance models. It is crucial to note that more severe diagnoses should not be taken directly but only after adjustment for their relative share.

However, there is an indirect method of detecting more severe infections, irrespective of coding or utilization differences, as there is a clear linkage with mortality. The overall number of influenza and lower respiratory infection visits is a strong predictor of subsequent respiratory deaths [7]. Thus, the epidemiological pattern for more severe but less monitored pathogens can be gauged based on well‐documented climatic differences in the annual cycle of mortality. Poland, as such, depicts a matching cycle of mortality typical for regions with a continental climate, which persists also in regions with harsher climates, although with an ever‐flatter amplitude as the infection season becomes longer but less intense [41, 42]. Conversely, in regions with a temperate climate like Great Britain, the amplitude is not only higher but also shapes differently, as the infection season is noticeably shorter [41, 43], while in warm Mediterranean regions, the long summer further interrupts the infection season, and the period of lowest mortality is delayed towards September [41, 44]. This proxy gives a good general guideline, except that a mortality‐based method would fail to detect early autumn rhinovirus infection spike that is primarily visible through numerous mild pediatric visits. This limitation could be overcome by considering studies from the Netherlands [11] and Great Britain [10] which implicitly suggest a delay of several weeks in the onset of this autumn wave of milder infection in temperate climate countries.

Additionally, there are factors that may lack universality, thus requiring adjustment to local conditions. Notably, the start of the school year in Poland on September 1 affects the admission of new children to nurseries, constrained by available space, modulating the early autumn infection wave. Secondly, there are 2‐week winter holidays rotated between provinces, occurring between Weeks 3 and 8. This interruption seems to impact the influenza wave among children, whereas it is not as noticeable among older age groups. Thirdly, there may be unique utilization patterns, such as free visits for patients under the single‐payer system, though the system becomes strongly capacity‐constrained during holidays or peak infection season. Additionally, Poland is among the European countries contending with a significant challenge of antibiotic overprescription [45, 46].

The results, beyond improving understanding, have direct practical applications. While there is strong variability between seasons, such as different timing and magnitude of influenza A waves or years without a noticeable influenza B wave [21, 22, 38], the observed mechanism is stochastic, and some patterns persist and could be utilized. Firstly, the model quite strongly shows a late summer period, which appears to be the most suitable moment for holidays for pediatric primary healthcare workers. The existence of a period of relatively lower infection prevalence in midautumn may serve as a highly suitable moment for pediatric influenza vaccination actions, both because of a lower share of ill kids and relatively quickly falling protection against symptomatic disease [47]. Conversely, late winter appears as least suitable period for vaccination in general due to likely increased number of cancellations and system overutilization.

## 5 Conclusion

Pediatric respiratory visits follow mostly stable annual pattern—after a late summer minimum, there is a sudden surge in mild infections in early September, followed by a slow decline in overall visits. Severity increases, with influenza peaks from January to March. A steady decline follows, except for a midsummer peak in tonsillopharyngitis.

## Author Contributions

**Marcin Piotr Walkowiak:** writing – original draft, investigation, conceptualization, validation, methodology, visualization, writing – review and editing, software, formal analysis, data curation. **Jarosław Walkowiak:** resources, supervision, funding acquisition. **Dariusz Walkowiak:** writing – review and editing, project administration, conceptualization.

## Ethics Statement

The authors have nothing to report.

## Conflicts of Interest

The authors declare no conflicts of interest.

### Peer Review

The peer review history for this article is available at https://www.webofscience.com/api/gateway/wos/peer‐review/10.1111/irv.70037.

## Supporting information


**Table S1.** Annual number of pediatric respiratory diagnoses (in thousands).

## Data Availability

Data are available on reasonable request.
