# Supplementary material for: Respiratory Diagnoses Year‐Round: Unraveling the Multifaceted Pediatric Infection Cycles
Source: Influenza Other Respir Viruses. 2024 Nov 4;18(11):e70037. doi: 10.1111/irv.70037 (PMC11534662; doi:10.1111/irv.70037)
Supplement: Supplementary file 1 — Table S1. Annual number of pediatric respiratory diagnoses (in thousands). [file IRV-18-e70037-s001.docx]

| **Year** | **J00** | **J01** | **J02** | **J03** | **J04** | **J05** | **J06** | **J11** | **J18** | **J20** | **J21** | **J22** | **Total** |
| --- | --- | --- | --- | --- | --- | --- | --- | --- | --- | --- | --- | --- | --- |
|  | Common cold | Sinusitis | Pharyngitis | Tonsillitis | Laryngotracheitis | Croup | Other upper | Influenza | Pneumonia | Bronchitis | Bronchiolitis | Other lower |  |
| **2010** | 4,343 | 350 | 2,068 | 1,036 | 586 | 12 | 2,456 | 40 | 327 | 1,357 | 8 | 63 | 12,646 |
| **2011** | 4,750 | 346 | 2,185 | 1,076 | 605 | 13 | 2,796 | 111 | 386 | 1,488 | 9 | 75 | 13,840 |
| **2012** | 4,967 | 369 | 2,105 | 1,135 | 636 | 13 | 3,000 | 59 | 458 | 1,570 | 10 | 95 | 14,417 |
| **2013** | 5,189 | 404 | 2,129 | 1,162 | 690 | 71 | 3,484 | 137 | 453 | 1,771 | 10 | 111 | 15,612 |
| **2014** | 5,050 | 398 | 2,033 | 1,144 | 662 | 111 | 3,502 | 51 | 421 | 1,839 | 10 | 115 | 15,337 |
| **2015** | 4,828 | 392 | 1,900 | 1,050 | 710 | 112 | 3,533 | 88 | 386 | 1,674 | 11 | 120 | 14,804 |
| **2016** | 4,753 | 365 | 1,743 | 940 | 826 | 94 | 3,529 | 104 | 369 | 1,484 | 12 | 128 | 14,347 |
| **2017** | 4,626 | 357 | 1,619 | 889 | 832 | 95 | 3,540 | 87 | 417 | 1,488 | 16 | 149 | 14,114 |
| **2018** | 4,439 | 343 | 1,546 | 851 | 810 | 87 | 3,497 | 114 | 402 | 1,352 | 16 | 160 | 13,618 |
| **2019** | 4,095 | 305 | 1,426 | 789 | 857 | 86 | 3,264 | 95 | 327 | 1,202 | 18 | 152 | 12,617 |

Table S1: Annual number of pediatric respiratory diagnoses (in thousands).
